# Parasites of reptiles in Iran (1922–2023): A literature review

**DOI:** 10.1016/j.ijppaw.2024.100992

**Published:** 2024-09-16

**Authors:** Alireza Sazmand, MohammadParsa Miadfar, Georgiana Deak, Mohammad Babaei, Jairo A. Mendoza-Roldan, Domenico Otranto

**Affiliations:** a Department of Pathobiology, Faculty of Veterinary Medicine, Bu-Ali Sina University, 6517658978, Hamedan, Iran; b Department of Parasitology and Parasitic Diseases, Faculty of Veterinary Medicine, University of Agricultural Sciences and Veterinary Medicine of Cluj-Napoca, 400372, Cluj-Napoca, Romania; c Department of Clinical Sciences, Faculty of Veterinary Medicine, Bu-Ali Sina University, 6517658978, Hamedan, Iran; d Department of Veterinary Medicine, University of Bari, 70010, Bari, Italy; e Department of Veterinary Clinical Sciences, City University of Hong Kong, Kowloon Tong, Hong Kong China

**Keywords:** Lizards, Pathogens, Reptiles, Snakes, Tortoise, Zoonoses

## Abstract

Reptiles are among the most diverse groups of animals, inhabiting nearly all continents and environments. Understanding their parasite biodiversity has garnered significant interest, particularly from a One Health perspective. Although the highly diverse reptile fauna of Iran, comprising 272 species i.e. 89 snakes (Serpentes), 171 lizards (Sauria), 8 turtles, 2 tortoises (Testudines), 1 crocodile (Crocodilia), and 1 worm-lizard (Amphisbaenia), there is a shortage of information about parasites. The present review is a compilation of 62 studies published from 1922 to August 2024. We present information on 56 species of reptiles from five groups (amphisbaenians, crocodiles, testudines, snakes, and lizards) and 98 parasitic taxa belonging to different protozoa and metazoa i.e. nematodes, cestodes, trematodes, acanthocephala, leeches, ticks, mites, and myiasis -producing flies. Although 63 taxa were diagnosed at the species level, 35 parasite taxa were only reported at the genus or family levels. Reviewing the literature, we found a paucity of information about endemic reptiles several of which are vulnerable species. Considering that some of the detected parasites e.g. *Cryptosporidium* and amoebae have serious clinical and/or public health threats molecular diagnostic techniques are needed for precise identification and understanding of the epidemiology and the potential zoonotic implications associated with parasites of reptiles. There is also a need to understand the exact distribution and host-parasite associations in different reptilian species present in Iran including the role of the reptiles as intermediate and reservoir hosts.

## Highlights

• The present review is a compilation of studies published from 1922 to August 2024.
• We present parasites reported from 56 species of reptiles.
• To date, 98 parasitic taxa belonging to different protozoa and metazoa have been reported.
• We found a paucity of information about native reptiles many of which are vulnerable species.

## Graphical abstract

**Figure:**
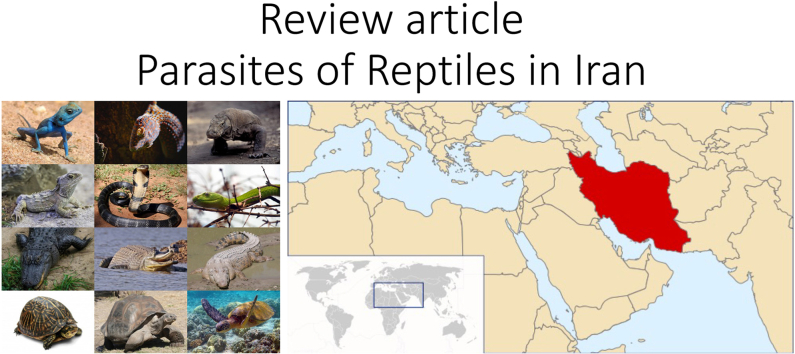

## 1 Background

Reptiles are among the most diverse groups of animals that inhabit almost all continents and environments (Roll et al., 2017). This class of animals includes about 1200 genera, and more than 11,000 species mainly within four orders i.e. Squamata (lizards, snakes, and worm-lizards), Testudines (turtles and tortoises), Crocodylia (land and sea crocodiles) and Rhynchocephalia (i.e., one species of tuataras namely *Sphenodon punctatus*) (Uetz et al., 2019). Like other animal groups, reptiles are hosts of viral, bacterial, and parasitic pathogens, some of which are of zoonotic concern (Mendoza-Roldan et al., 2023a, Mendoza-Roldan et al., 2023b). Reptiles live in and around human habitats, they are used as important sources of food, medicines, and materials (e.g., the leather industry) in some parts of the world, and become increasingly common domestic pets (Mendoza-Roldan et al., 2020). Hence, understanding the biodiversity of their parasites has become of great interest among researchers, especially from a *One Health* perspective, since reptiles are host to a range of potentially zoonotic parasites of many different taxa i.e. *Alaria* (trematoda), *Spirometra* (cestoda), *Anisakis*, *Eustrongylides*, *Angiostrongylus*, *Gnathostoma* and *Trichinella* (nematoda), *Armillifer* (pentastomida), *Ophionyssus natricis* (acari), *Trypanosoma*, *Sarcocystis*, *Cryptosporidium* (protozoa) (Leung, 2024; Mendoza-Roldan et al., 2023).

Iranian reptiles are the largest group of vertebrates living in the country. According to the latest official reports, the reptile fauna of Iran comprised 272 species i.e. 89 snakes (Serpentes), 171 lizards (Sauria), 8 turtles, 2 tortoises (Testudines), 1 crocodile (Crocodilia), and 1 worm-lizard (Amphisbaenia) (Kasfash et al., 2020; Mozaffari et al., 2016; Safaei-Mahroo, 2019; Šmíd et al., 2014). However, as a result of the advancement of molecular methods, there are growing pieces of evidence regarding a wider diversity (Mozaffari et al., 2020). To date (September 12, 2024), 213 Iran reptile species have been recorded in the International Union for Conservation of Nature and Natural Resources (IUCN) Red List of Threatened Species (www.iucnredlist.org) out of which 2 are Critically Endangered (CE), 4 Endangered (EN), 9 Vulnerable (VU), and 9 Near Threatened (NT); several being endemic in the Country (e.g. Iranian Mountain Steppe Viper *Vipera ebneri*, *Montivipera albicornuta*, *Montivipera latifii*).

Since so far, most available studies on reptiles in Iran have focused on ecological investigations of hosts and conservational aspects rather than on the parasites (Kafash et al., 2020; Zare Khormizi et al., 2021), this study aimed to review the published research on parasites of reptiles in Iran from 1931 to August 2024.

## 2 Methods

We checked all available documents on each of the search terms which included a combination of Iran or Iranian (in Persian, English, and French) with each of the words “snake”, “lizard”, “turtle”, “tortoise”, “reptile” and generic names of the parasites of reptiles extracted from different sources e.g. “Flynn's Parasites of Laboratory Animals” (Mitchell, 2007), “parasites in pet reptiles” (Rataj et al., 2011) and “zoonotic parasites of reptiles: a crawling threat” (Mendoza-Roldan et al., 2020). The databases and search engines employed for the present literature review were PubMed (https://pubmed.ncbi.nlm.nih.gov/), Google Scholar (https://scholar.google.com/), Scientific Information Database of Iran (www.sid.ir), the collection of defended theses at all Iranian universities (https://irandoc.ac.ir/) and the collection of proceedings of Iranian scientific congresses on veterinary medicine, animal science and parasitology (https://civilica.com/). Databases were last visited on September 12, 2024.

In total, 62 documents, comprising 61 articles in peer-reviewed journals and one article presented at a national conference, were found and included. The literature was extracted from Google Scholar (*n* = 35), Pubmed (*n* = 35), and Civilica (*n* = 1). No defended thesis with a focus on parasites of reptiles was found. In regards to the language, two were written in Persian, four in French, and 56 in English.

Valid names of the reported scientific names (both parasites and reptiles) in older literature were obtained from updated resources i.e. http://www.reptile-database.org/and https://www.gbif.org/.

## 3 Results and discussion

### 3.1 Protozoan infections

#### 3.1.1 Blood parasites

Infections with protozoan parasites of blood are very common in reptiles globally. Hemoparasites of reptiles are included in five groups of 1. Plasmodiids (*Plasmodium*, *Haemocystidium*, *Saurocytozoon*, *Fallisia*, *Haemoproteus*, *Progarnia*), 2. Hemogregarines (*Haemogregarina*, *Hepatozoon*, *Hemolivia*, *Karyolyses*), 3. Hemococcidia (*Schellackia*, *Lainsonia, Lankestrella*), 4. Kinetoplastids (*Trypanosoma*, *Sauroleishmania*), 5. Piroplasmids (*Sauroplasma*, *Serpentoplasma*) (Telford, 2009). Many of these hemoparasites are morphologically similar to related protozoa of other vertebrates. While most infections are asymptomatic, heavy infections may result in anaemia (Mitchell, 2007). Furthermore, filarial larvae (Nematoda), in addition to rickettsial, chlamydial, and viral parasites of blood cells have frequently been found in snakes, chelonians, and lizards (Telford, 2009; Ursula et al., 2014). Common to these parasites is that they need arthropods or leeches as vectors for their development or transmission (Mitchell, 2007). In Iran so far, *Hepatozoon* spp., *Leishmania*/*Sauroleishmania* spp., *Serpentoplasma* spp., *Aegyptianella* spp., *Hemolivia mauritanica,* and microfilariae of *Oswaldofilaria chlamydosauri* have been investigated in the blood of reptiles by light microscopy (Javanbakht et al., 2015b; Kazemi et al., 2004; Molavi et al., 2018; Sajjadi and Javanbakht, 2017). One study recorded intraerythrocytic haemoparasites without sequential data or genus designation (Nasiri et al., 2014). There is only one PCR-based study in which *Haemoproteus anatolicum* and *Haemoproteus caucasica* were identified in tortoises (Javanbakht et al., 2015a) (Table 1). There is little known about vectors of reptiles’ hemoparasites in Iran. So far in only one study the role of the hard tick *Hyalomma aegyptium* in the transmission of *Hemolivia mauritanica* has been shown (Javanbakht et al., 2015b).

**Table 1:** Haemoparasites of reptiles reported from Iran.

Parasite	Host	Common name	Reference
*Leishmania* sp.	*Paralaudakia caucasia* (Eichwald, 1831). Reported as *Agama caucasica*	Caucasian agama	(Kazemi et al., 2004, 2004b; Nadim et al., 1968a; b; Rashti et al., 1971)
	*Mesalina martini* (Boulenger, 1897). Reported as *Eremias guttulata*	Martin's desert racer	
	*Heremites auratus* Linnaeus, 1758. Reported as *Mabuya aurata*	Golden grass skink	
	*Trapelus agilis* (Olivier, 1807). Reported as *Agama agilis*	Brilliant ground agama	
	*Phrynocephalus scutellatus* (Olivier, 1807). Reported as *Phrynocephalus seatellatua*	Gray toad head agama	
	*Laudakia melanura* Blyth, 1854. Reported as *Agama melanura*	Black agama	
	*Tenuidactylus caspius* (Eichwald, 1831). Reported as *Cyrtopodion caspius*	Caspian bent-toed gecko	
*Leishmania gymnodactyli*	*Tenuidactylus caspius* (Eichwald, 1831). Reported as *Cyrtopodion caspius*	Caspian bent-toed gecko	Seyedi Rashti et al. (1994)
*Haemogregarina stepanowi* Danilewsky, 1885	*Mauremys capsica* (Gmelin, 1774)	Caspian pond turtle	(Dvořáková et al., 2014; Javanbakht and Sharifi, 2014)
	*Emys orbicularis* (Linnaeus, 1758)	European pond turtle	
*Haemogregarina* sp.	*Mauremys capsica* (Gmelin, 1774)	Caspian pond turtle	Rakhshandehroo et al. (2016)
*Haemocystidium grahami*Shortt (1922)	*Laudakia nupta* (De Filippi, 1843)	Large-scaled rock agama	Shortt (1922)
*Haemocystidium phyllodactyli*Shortt (1922)	*Asaccus elisae* (Werner, 1895). Reported as *Phyllodactylus elisae*	Elisa's leaf-toed gecko	Shortt (1922)
*Haemoproteus anatolicum* Orkun and Guven 2013	*Testudo hermanni* Gmelin, 1789. Reported as *Testudo graeca*	Hermann's tortoise	(Javanbakht et al., 2015a)
*Haemoproteus caucasica* Krasilnikov, 1965	*Testudo horsfieldii* Gray, 1844	Central Asian tortoise	(Javanbakht et al., 2015a)
	*Testudo hermanni* Gmelin, 1789. Reported as *Testudo graeca*	Hermann's tortoise	
*Hemolivia mauritanica* (Sergent and Sergent, 1904)	*Testudo horsfieldii* Gray, 1844	Central Asian tortoise	Javanbakht et al. (2015b)
	*Testudo hermanni* Gmelin, 1789. Reported as *Testudo graeca*	Hermann's tortoise	
*Rickettsia* sp. (Probably *Aegyptianella)*	*Natrix natrix* (Linnaeus, 1758)	European grass snake	Sajjadi and Javanbakht (2017)
	*Natrix tessellata* (Laurenti, 1768)	Dice snake	
*Serpentoplasma* sp.	*Natrix tessellata* (Laurenti, 1768)	Dice snake	Sajjadi and Javanbakht (2017)
*Hepatozoon ophisauri* (Tartakovskii, 1913)	*Pseudopus apodus* (Pallas, 1775)	European glass lizard	Zechmeisterová et al. (2021)
*Hepatozoon colubrid* (Börner, 1901)	*Zamenis longissimus* (Laurenti, 1768)	Aesculapian snake	Zechmeisterová et al. (2021)
*Hepatozoon* sp.	*Macrovipera lebetinus* subsp. *Obtusa* (Dwigubsky, 1832). Reported as *Macrovipera lebetina obtuse*	West-Asian blunt-nosed viper	Junsiri et al. (2024)
	*Cerastes gasperettii* Leviton and Anderson, 1967	Arabian horned viper	Rashnavadi (2012)
	*Zamenis longissimus* (Laurenti, 1768)	Aesculapian snake	
*Oswaldofilaria chlamydosauri* (Breinl, 1913). **Microfilariae**. Reported as *Oswaldofilaria chlamydosauri* (Breinl, 1912)	*Paralaudakia caucasia* (Eichwald, 1831)	Caucasian agama	Molavi et al. (2018)
*Karyolysus* sp.	*Darevskia chlorogaster* (Boulenger, 1908)	Green Bellied lizard	Javanbakht et al. (2022)
*Lankestrella* sp.	*Eremias persica* Blanford, 1875	Persian race-runner	Javanbakht and Hajiyan (2024)
	*Ophisops elegans*Mendoza-Roldan et al., 2021	Snake-eyed lizard	
*Schellackia* sp.	*Darevskia chlorogaster* (Boulenger, 1809)	Greenbelly lizard	Zechmeisterová et al. (2021)
	*Lacerta strigata* (Eichwald, 1831)	Caspian green lizard	
Intraerythrocytic haemoparasites	*Pseudocerastes persicus* (Duméril, Bibron & Duméril, 1854)	Persian horned viper	Nasiri et al. (2014)
	*Macrovipera lebetinus* subsp. *Obtusa* (Dwigubsky, 1832). Reported as *Macrovipera lebetina* subsp. *Obtusa*	West-Asian blunt-nosed viper	
	*Naja siamensis* Laurenti, 1768. Reported as *Naja oxiana*	Central Asian cobra	
	*Dolichophis caspius* (Gmelin, 1789). Reported as *Coluber caspius*	Caspian whipsnake	

*Leishmania gymnodactyli* was detected in *Sergentomyia sintoni* (Seyedi Rashti et al., 1994). Furthermore, *Sergentomyia clydei* and *Sergentomyia dentata* have been recognized as vectors of lizard leishmaniasis (Yaghoobi-Ershadi, 2012). Snakes and lizards contribute to the distribution of several *Leishmania* species pathogenic for humans such as *Leishmania tropica* the causative agent of cutaneous leishmaniasis (CL) (Zhang et al., 2016). Although CL is widely distributed in 25 of 31 Iranian provinces with an annual incidence of 30.9 per 100,000 in the Iranian population (Holakouie-Naieni et al., 2017) the role of reptiles in the epidemiology of CL in the country warrants further investigations.

#### 3.1.2 Gastrointestinal protozoa

Reptiles are hosts for various flagellates (e.g. *Giardia* spp., *Hexamita* spp., *Trichomonas* spp.), amoebae (e.g. *Entamoeba invadens*), coccidia (e.g. *Eimeria* spp., *Isospora* spp., *Cryptosporidium* spp., *Blastocystis* spp.) and ciliates (e.g. *Balantidium* spp., *Nyctotheroides* spp.) (Greiner, 2003; Mitchell, 2007). Infection with these parasites may cause enteritis, weight loss, regurgitation, colitis, gastritis, and death (Wilson and Carpenter, 1996). In Iran, gastrointestinal protozoan parasites were studied in two studies in which *Eimeria* spp., *Isospora* spp., *Cryptosporidium* spp., *Balantidium* spp., *Nyctotheroides* spp., *Cyclospora* spp., trichomonads, and amoebae were recorded (Arabkhazaeli et al., 2018; Nasiri et al., 2014) (Table 2). Among the reported protozoan parasites *Cryptosporidium* spp. Has a potential risk for human infections (Díaz et al., 2013; Mendoza-Roldan et al., 2020, 2023, 2024; Traversa et al., 2008).

**Table 2:** Protozoan parasites of reptiles reported from Iran.

Parasite	Host	Common name	Reference
*Toxoplasma gondii* (Nicolle and Manceaux, 1908)	*Naja oxiana* (Eichwald, 1831)	Central Asian cobra	Nasiri et al. (2016)
	*Pseudocerastes fieldi* Schmidt, 1930. Reported as *Pseudocerastes persicus fieldi*	Field's horned viper	
	*Montivipera raddei* (Boettger, 1890). Reported as *Vipera albicornuta*	Caucasus viper	
	*Gloydius intermedius* (Strauch, 1868). Reported as *Agkistrodon intermedius caucasicus*	Caucasian pit viper	
	*Macrovipera lebetinus* subsp*. Obtusa* (Dwigubsky, 1832). Reported as *Vipera lebetina obtusa*	West-Asian blunt-nosed viper	
*Neospora caninum* Dubey et al., 1988	**Snakes**		
	*Pseudocerastes fieldi* Schmidt, 1930. Reported as *Pseudocerastes persicus fieldi*	Field's horned viper	Nasiri and Jameie (2023)
	*Naja oxiana* (Eichwald, 1831)	Central Asian cobra	Nasiri and Jameie (2023)
	*Montivipera raddei* (Boettger, 1890). Reported as *Vipera albicornuta*	Caucasus viper	Nasiri and Jameie (2023)
*Eimeria* sp.	**Snakes**		
	*Montivipera raddei* (Boettger, 1890)	Caucasus viper	Nasiri et al. (2014)
	*Vipera eriwanensis* subsp. *Eriwanensis* (Reuss, 1933). Reported as *Vipera ursinii eriwanensis*	Alburzi viper	Nasiri et al. (2014)
	*Python molurus* (Linnaeus, 1758)	Indian rock python	Arabkhazaeli et al. (2018)
	**Lizards**		
	*Pogona vitticeps* (Ahl, 1926)	Central bearded dragon	Arabkhazaeli et al. (2018)
*Blastocystis* sp.	**Snakes**		
	*Macrovipera lebetinus* subsp. *Obtusa* (Dwigubsky, 1832). Reported as *Macrovipera lebetina* subsp. *Obtusa*	West-Asian blunt-nosed viper	Nasiri et al. (2014)
Trichomonads	**Snakes**		
	*Eryx jaculus* (Linnaeus, 1758)	Javelin sand boa	Arabkhazaeli et al. (2018)
	**Lizards**		
	*Iguana iguana* (Linnaeus, 1758)	Common green iguana	Arabkhazaeli et al. (2018)
*Cryptosporidium* sp.	**Snakes**		
	*Echis carinatus* subsp. *Sochureki* Stemmler, 1969	Phoorsa	Arabkhazaeli et al. (2018)
	*Ophiophagus hannah* (Cantor, 1836)	King cobra	Arabkhazaeli et al. (2018)
	*Macrovipera lebetinus* subsp. *Obtusa* (Dwigubsky, 1832). Reported as *Macrovipera lebetina* subsp. *Obtusa*	West-Asian blunt-nosed viper	Arabkhazaeli et al. (2018)
	**Lizards**		
	*Iguana iguana* (Linnaeus, 1758)	Common green iguana	Arabkhazaeli et al. (2018)
	*Tiliqua scincoides* (White, 1790)	Bluetongued lizard	Arabkhazaeli et al. (2018)
	*Pogona vitticeps* (Ahl, 1926)	Central bearded dragon	Arabkhazaeli et al. (2018)
	**Tortoises**		
	*Testudo horsfieldii* Gray, 1844	Central Asian tortoise	Arabkhazaeli et al. (2018)
*Balantidium* sp.	**Lizards**		
	*Iguana iguana* (Linnaeus, 1758)	Common green iguana	Arabkhazaeli et al. (2018)
	**Tortoises**		
	*Testudo horsfieldii* Gray, 1844	Central Asian tortoise	Arabkhazaeli et al. (2018)
Amoebae	**Lizards**		
	*Iguana iguana* (Linnaeus, 1758)	Common green iguana	Arabkhazaeli et al. (2018)
	*Eublepharis macularius* (Blyth, 1854)	Leopard gecko	Arabkhazaeli et al. (2018)
	**Tortoises**		
	*Testudo horsfieldii* Gray, 1844	Central Asian tortoise	Arabkhazaeli et al. (2018)
*Cyclospora* sp.	**Lizards**		
	*Tiliqua scincoides* (White, 1790)	Bluetongued lizard	Arabkhazaeli et al. (2018)
*Isospora* sp.	**Lizards**		
	*Pogona vitticeps* (Ahl, 1926)	Central bearded dragon	Arabkhazaeli et al. (2018)
	**Turtles**		
	*Trachemys scripta* subsp. *Elegans* (Wied, 1838)	Pondslider	Arabkhazaeli et al. (2018)
*Hexamita batrachorum* Swezy, 1915	**Snakes**		
	*Pseudocerastes fieldi* Schmidt, 1930	Field's horned viper	Nasiri et al. (2014)
	*Naja oxiana* (Eichwald, 1831)	Central Asian cobra	Nasiri et al. (2014)
	*Montivipera raddei* (Boettger, 1890)	Caucasus viper	Nasiri et al. (2014)
	*Macrovipera lebetinus* subsp. *Obtusa* (Dwigubsky, 1832). Reported as *Macrovipera lebetina* subsp. *Obtusa*	Levant viper	Nasiri et al. (2014)
	*Vipera eriwanensis* subsp. *Eriwanensis* (Reuss, 1933). Reported as *Vipera ursinii eriwanensis*	Alburzi viper	Nasiri et al. (2014)
	*Gloydius intermedius* (Strauch, 1868). Reported as *Agkistrodon intermedius caucasicus*	Caucasian pit viper	Nasiri et al. (2014)
*Nyctotheroides* sp.	**Snakes**		
	*Pseudocerastes fieldi* Schmidt, 1930. Reported as *Pseudocerastes persicus fieldi*	Field's horned viper	Nasiri et al. (2014)

##### 3.1.2.1 Toxoplasma gondii

In one study molecular analysis identified the presence of *Toxoplasma gondii* DNA in the brain of 55 individual snakes from five different species i.e. *Pseudocerastes fieldi* Schmidt, 1930, *Naja siamensis* Laurenti, 1768, *Montivipera raddei* (Boettger, 1890), *Macrovipera lebetina* subsp. *Obtusa* (Dwigubsky, 1832) and *Gloydius intermedius* (Strauch, 1868; Nasiri et al., 2016). *Toxoplasma gondii* is an obligate intercellular protozoan parasite that can infect a wide range of warm- and cold-blooded animals as well as humans (Długońska, 2017). Due to its exceptionally wide range of warm- and cold-blooded hosts, *Toxoplasma gondii* is one of the most successful zoonotic parasites on the earth (Djurković-Djaković et al., 2019). Considering that in two studies that detected DNA of the parasite in tissues of snakes (Nasiri et al., 2016) (Anah and Al-Mayali, 2018) isolation of viable parasites was not attempted, the role of the reptiles in the epidemiology of toxoplasmosis remains dubious.

### 3.2 Helminthoses

Reptiles have important roles as both definitive and intermediate hosts of helminths (Carbonara et al., 2023; Šlapeta et al., 2017). Nematodes are among the most common parasites identified in reptiles worldwide (Bower et al., 2019; Kuśmierek et al., 2020), including in Iran where several studies were done highlighting the species diversity, prevalence, and potential pathological effects of these parasites on their hosts. Investigations on this topic date back to 1966 when Petter (1966) provided data about nematode taxonomy in reptiles. In 2004, nematodes of the European pond turtle were mentioned as part of a larger study investigating the pond culture of sturgeon (Pazooki and Aghaeei Moghadam, 2004). In further years, several studies were performed either specifically on reptiles or as part of a larger study that investigated nematodes of snakes, including venomous ones and lizards (Table 3). Nematode infection in Iranian reptiles indicates significant prevalence and a wide diversity of species, some of which e.g. *Anisakis* and *Ophidascaris* are likely to pose a considerable health risk to hosts (Hossain et al., 2023; Magnino et al., 2009).

**Table 3:** Helminths of reptiles reported from Iran.

Parasite	Host species	Common name	Reference
**Trematoda**			
*Telorchis assula* (Dujardin, 1845)	*Natrix natrix* (Linnaeus, 1758)	European grass snake	(Halajian et al., 2013; Nasiri et al., 2014; Shayegh et al., 2016; Yossefi et al., 2014; Yousesfi et al., 2013)
	*Natrix tessellata* (Laurenti, 1768)	Dice snake	
	*Emys orbicularis* (Linnaeus, 1758)	European pond turtle	
	*Mauremys caspica* (Gmelin, 1774)	Caspian turtle	
*Telorchis* sp.	*Mauremys caspica* (Gmelin, 1774)	Caspian turtle	Youssefi et al. (2016)
**Cestoda**			
*Ophiotaenia europaea* Odening, 1963	*Natrix natrix* (Linnaeus, 1758)	European grass snake	(Halajian et al., 2013; Yossefi et al., 2014; Youssefi et al., 2010)
	*Natrix tessellata* (Laurenti, 1768)	Dice Snake	
*Ophiotaenia* sp.	*Natrix natrix* (Linnaeus, 1758)	European grass snake	Nasiri et al. (2014)
*Oochoristica fibrata* Meggitt, 1927	*Boiga trigonata* subsp. *melanocephala* (Annandale, 1904)	Indian gamma snake	Mariaux et al. (2017)
*Oochoristica tuberculata* (Rudolphi, 1819)	*Varanus griseus* (Daudin, 1803)	Desert monitor	(Dollfus, 1965; Goldberg and Bursey, 2010)
	*Ophisops elegans*Mendoza-Roldan et al., 2021	Snake-eyed lizard	
*Oochoristica* sp.	*Platyceps rhodorachis* (Jan 1865). Reported as *Zamenis rhodorhachis*	Aesculapian snake	Dollfus (1965)
	*Malpolon monspessulanus* subsp. *insignitus*	Montpellier snake	
*Nematotaenia dispar* (Goeze, 1782)	*Varanus griseus* (Daudin, 1803)	Desert monitor	Dollfus (1965)
*Nematotaenia* sp.	*Varanus griseus* (Daudin, 1803)	Desert monitor	Dollfus (1965)
*Joyeuxiella echinorhynchoides* (Sonsino, 1884) **cysticercoid**	*Platyceps najadum* (Eichwald, 1831). Reported as *Coluber najadum*	Dahl's whip snake	Dollfus (1965)
**Nematoda**			
*Entomelas entomelas* (Dujardin, 1845)	*Pseudopus apodus* (Pallas, 1775)	European glass lizard	Halajian et al. (2013)
*Rhabdias fuscovenosa* (Railliet, 1899)	*Natrix natrix* (Linnaeus, 1758)	European grass snake	(Halajian et al., 2013; Yossefi et al., 2014)
	*Natrix tessellata* (Laurenti, 1768)	Dice snake	
Ascarididae	*Naja oxiana* (Eichwald, 1831)	Central Asian cobra	Nasiri et al. (2014)
*Kalicephalus viperae* (Rudolphi, 1819)	*Macrovipera lebetinus* (Linnaeus, 1758)	Levantine viper	Larki et al. (2023)
*Abbreviata kazachstanica*Mariaux et al., 2017	*Pseudopus apodus* (Pallas, 1775)	European glass lizard	Ghanbari Johkool et al. (2021)
*Ophidascaris filaria* (Dujardin, 1845)	*Python molurus* (Linnaeus, 1758)	Indian python	Ganjali et al. (2015)
*Skrjabinodon pigmentatus* (Mariaux et al., 2017)	*Laudakia caucasia* (Eichwald, 1831)	Caucasian agama	Rezazadeh et al. (2012)
*Spauligodon lacertae* Sharpilo, 1966	*Laudakia caucasia* (Eichwald, 1831)	Caucasian agama	Rezazadeh et al. (2012)
*Thelandros baylisi* (Chabaud, 1953)	*Laudakia caucasia* (Eichwald, 1831)	Caucasian agama	Rezazadeh et al. (2012)
*Abbreviata baltazardi* (Chabaud 1953)	*Phrynocephalus helioscopus* (Pallas, 1771)	Sunwatcher toadhead agama	Chabaud (1953)
*Oswaldofilaria chlamydosauri* (Breinl, 1913). Reported as *Oswaldofilaria chlamydosauri* (Breinl, 1912) **microfilariae**	*Paralaudakia caucasia* (Eichwald, 1831)	Caucasian agama	Molavi et al. (2018)
*Thelandros agama* Adamson and Nasher, 1984	*Laudakia nupta* (De Filippi, 1843)	Large-scaled rock agama	Rahimian et al. (2014)
*Thelandros popovi* (Mariaux et al., 2017)	*Laudakia nupta* (De Filippi, 1843)	Large-scaled rock agama	Rahimian et al. (2014)
*Thelandros karkasensis*Rahimian et al., 2014	*Laudakia nupta* (De Filippi, 1843)	Large-scaled rock agama	Rahimian et al. (2014)
*Thubunea* sp.	*Laudakia nupta* (De Filippi, 1843)	Large-scaled rock agama	Rahimian et al. (2014)
*Parapharyngodon thulini*Rahimian et al., 2014	*Laudakia nupta* (De Filippi, 1843)	Large-scaled rock agama	Rahimian et al. (2014)
*Alaeuris numidica* (Seurat, 1918)	*Testudo graeca zarudnyi* Nikolsky, 1896	Iranian tortoise	Petter (1966)
*Mehdiella microstoma* (Drasche, 1884)	*Testudo graeca zarudnyi* Nikolsky, 1896	Iranian tortoise	Petter (1966)
	*Testudo horsfieldii* Gray, 1844	Central Asia tortoise	
*Mehdiella longissimi* Petter (1966)	*Testudo horsfieldii* Gray, 1844	Central Asia tortoise	Petter (1966)
*Mehdiella stylosa* Thapar, 1925	*Testudo graeca zarudnyi* Nikolsky, 1896	Iranian tortoise	Petter (1966)
	*Testudo horsfieldii* Gray, 1844	Central Asia tortoise	
*Mehdiella uncinata* Drasche, 1884	*Testudo graeca zarudnyi* Nikolsky, 1896	Iranian tortoise	Petter (1966)
	*Testudo horsfieldii* Gray, 1844	Central Asia tortoise	
*Tachygonetria conica* (Drasche, 1884)	*Testudo graeca zarudnyi* Nikolsky, 1896	Iranian tortoise	Petter (1966)
	*Testudo horsfieldii* Gray, 1844	Central Asia tortoise	
*Tachygonetria dentata* (Drasche, 1884)	*Testudo graeca zarudnyi* Nikolsky, 1896	Iranian tortoise	Petter (1966)
	*Testudo horsfieldii* Gray, 1844	Central Asia tortoise	
*Tachygonetria longicollis* (Schneider, 1866)	*Testudo graeca zarudnyi* Nikolsky, 1896	Iranian tortoise	Petter (1966)
	*Testudo horsfieldii* Gray, 1844	Central Asia tortoise	
*Tachygonetria palearcticus* Seurat, 1918	*Testudo graeca zarudnyi* Nikolsky, 1896	Iranian tortoise	Petter (1966)
	*Testudo horsfieldii* Gray, 1844	Central Asia tortoise	
*Tachygonetria pusilla* Seurat, 1918	*Testudo graeca zarudnyi* Nikolsky, 1896	Iranian tortoise	Petter (1966)
	*Testudo horsfieldii* Gray, 1844	Central Asia tortoise	
*Tachygonetria robusta* (Drasche, 1884)	*Testudo graeca zarudnyi* Nikolsky, 1896	Iranian tortoise	Petter (1966)
	*Testudo horsfieldii* Gray, 1844	Central Asia tortoise	
*Thaparia thapari* (Dubinina, 1949)	*Testudo graeca zarudnyi* Nikolsky, 1896	Iranian tortoise	Petter (1966)
	*Testudo horsfieldii* Gray, 1844	Central Asia tortoise	
*Atractis baltazardi* Petter (1966)	*Testudo graeca zarudnyi* Nikolsky, 1896	Iranian tortoise	Petter (1966)
*Falcaustra araxiana* Massino, 1924	*Emys orbicularis* (Linnaeus, 1758)	European pond turtle	(Rajabloo et al., 2017; Shayegh et al., 2016)
*Serpinema microcephalus* (Dujardin, 1845)	*Emys orbicularis* (Linnaeus, 1758)	European pond turtle	(Hoseini et al., 2015; Shayegh et al., 2016; Youssefi et al., 2016)
	*Mauremys caspica* (Gmelin, 1774)	Caspian turtle	
*Falcaustra armenica* Massino, 1924	*Mauremys caspica* (Gmelin, 1774)	Caspian turtle	Youssefi et al. (2016)
Oxyuridae	*Mauremys caspica* (Gmelin, 1774)	Caspian turtle	Youssefi et al. (2016)
*Hysterothylacium* sp.	*Emys orbicularis* (Linnaeus, 1758)	European pond turtle	Pazooki and Aghaeei Moghadam (2004)
*Camallanus* sp.	*Emys orbicularis* (Linnaeus, 1758)	European pond turtle	Pazooki and Aghaeei Moghadam (2004)
*Anisakis* sp.	*Emys orbicularis* (Linnaeus, 1758)	European pond turtle	Pazooki and Aghaeei Moghadam (2004)
*Falcaustra ararath* (Massino, 1924)	*Mauremys caspica* (Gmelin, 1774)	Caspian turtle	Rakhshandehroo et al. (2020)
*Spiroxys* sp.	*Mauremys caspica* (Gmelin, 1774)	Caspian turtle	Rakhshandehroo et al. (2020)
Strongylid egg	*Iguana iguana* (Linnaeus, 1758)	Common green iguana	Arabkhazaeli et al. (2018)
	*Trapelus agilis* (Olivier, 1807)	Brilliant ground agama	
	*Pogona vitticeps* (Ahl, 1926)	Central bearded dragon	
	*Coluber nummifer* Reuss, 1834	Asian racer	
	*Testudo horsfieldii* Gray, 1844	Central Asia tortoise	
Oxyurid egg	*Iguana iguana* (Linnaeus, 1758)	Common green iguana	(Arabkhazaeli et al., 2018; Youssefi et al., 2016)
	*Eublepharis macularius* (Blyth, 1854)	Common leopard gecko	
	*Lacerta media* Lantz and Cyrén, 1920	Medium lizard	
	*Pogona vitticeps* (Ahl, 1926)	Central bearded dragon	
	*Macrovipera lebetinus* subsp. *Obtusa* (Dwigubsky, 1832). Reported as *Macrovipera lebetina* subsp. *Obtusa*	Levant viper	
	*Testudo horsfieldii* Gray, 1844	Central Asia tortoise	
	*Mauremys caspica* (Gmelin, 1774)	Caspian terrapin	
*Strongyloides* egg	*Tiliqua scincoides* (White, 1790)	Common bluetongue	(Arabkhazaeli et al., 2018; Nasiri et al., 2014)
	*Macrovipera lebetinus* subsp. *Obtusa* (Dwigubsky, 1832). Reported as *Macrovipera lebetina* subsp. *Obtusa*	Levant viper	
	*Ophiophagus hannah* (Cantor, 1836)	Hamadryad	
	*Naja oxiana* (Eichwald, 1831)	Central Asian cobra	
	*Vipera eriwanensis* subsp. *Eriwanensis* (Reuss, 1933). Reported as *Vipera ursinii eriwanensis*	Alburzi viper	
**Acanthocephala**			
*Centrorhynchus corvi* Fukui, 1929	*Naja oxiana* (Eichwald, 1831)	Central Asian cobra	Nasiri et al. (2014)
	*Montivipera raddei* (Boettger, 1890)	Caucasus viper	
	*Macrovipera lebetinus* subsp. *Obtusa* (Dwigubsky, 1832). Reported as *Macrovipera lebetina* subsp. *Obtusa*	West-Asian blunt-nosed viper	
	*Coluber caspius* Gmelin in Linnaeus, 1789	European whip snake	
**Leech**			
*Haementeria costata* (Müller, 1846). Also reported as *Placobdella costata*	*Emys orbicularis* (Linnaeus, 1758)	European pond turtle	(Hojjati et al., 2003; Kami et al., 2006)

Cestodes, on the other hand, can infect various reptiles with different pathological effects on the affected animals. In Iran, the European grass snakes (*Natrix natrix*) as well as the European glass lizard (*Pseudopus apodus*) are hosts for *Ophiotaenia* spp. (Nasiri et al., 2014). The first mention of cestodes in Iranian reptiles dates back to 1965 (Dollfus, 1965) with a publication that provided some detailed descriptions and classifications of various cestode species (Table 3). More recently, Mariaux et al. (2017) investigated the cestode diversity of reptiles with a highlight on the need for improved taxonomic and molecular approaches for the identification of different cestode species.

Trematodes are common helminths of reptiles with many species being reported at a global level (Bower et al., 2019). However, in Iran, few studies investigated the infection of reptiles by trematodes, and they include only snakes and turtles as investigated hosts (Halajian et al., 2013; Nasiri et al., 2014; Shayegh et al., 2016; Yossefi et al., 2014; Yousesfi et al., 2013, 2016). A total of 87 individual grass snakes (*Natrix natrix*) and dice snakes (*Natrix tessellata*) were investigated in three studies in Iran and an important proportion of them was infected by *Telorchis assula* (Halajian et al., 2013; Nasiri et al., 2014b; Yossefi et al., 2014), underlying the lack of data on this topic. The same trematode species, *Telorchis assula* was identified in the European pond turtle (*Emys orbicularis*) from Fars province with a high prevalence (48.07%) (Shayegh et al., 2016) suggesting a potential role as reservoir hosts for other reptiles. Additionally, the Caspian turtle (*Mauremys caspica*) was also found infected by *Telorchis* spp. (Youssefi et al., 2016). Although several trematodes are known to infect reptiles (Hughes et al., 1942), only *Telorchis assula* was reported in Iranian reptiles highlighting the need for further research to understand the real diversity of species as well as the impact of these infections on reptilian health.

Finally, acanthocephalans, generally known as thorny-headed helminths, can infect various hosts, including reptiles (Smales, 2014), and have severe health implications, like malnutrition and even death (Nasiri et al., 2014). In Iran, few studies have investigated the occurrence of acanthocephalans in local reptiles (Table 3). In snakes, larval stages of *Centrorhynchus corvi* were identified in the intestinal wall of seven snakes, associated with local necrosis (Nasiri et al., 2014b).

### 3.3 Ectoparasites

Parasitic arthropods of reptiles are important due to their role as parasites, but mainly because of their vectorial competence for various diseases (Mendoza-Roldan et al., 2021). In Iran, ticks and mites were mainly investigated in reptiles (Table 4). The first knowledge in Iran about ectoparasites in reptiles was documented (Baltazard et al., 1955; Vercammen-Grandjean et al., 1970) which identified several mite and tick species and provided some information on this topic.

**Table 4:** Ectoparasites of reptiles reported from Iran.

Parasite	Host species	Common name	Reference
**Ticks**			
*Ornithodoros tartakovskyi* Olenev, 1931	Testudine (French word: tortues)		Baltazard et al. (1955)
*Ornithodoros erraticus* Clifford, Kohls & Sonenshine, 1964	Testudine (French word: tortues)		Baltazard et al. (1955)
*Hyalomma aegyptium* Linnaeus, 1758	*Testudo* sp.	NS	(Abbassian-Lintzen, 1960; Banafshi et al., 2018)
	Lizard	NS	Abbassian-Lintzen (1960)
	*Testudo graeca* Linnaeus, 1758	Mediterranean spur-thighed tortoise	(Adeli-Sardou et al., 2019; Banafshi et al., 2018; Javanbakht et al., 2015b; Nabian and Mirsalimi, 2002; Razmjo et al., 2013; Tavassoli et al., 2007)
	*Laudakia nupta* (De Filippi, 1843)	Large-scaled rock agama	Razmjo et al. (2013)
	*Trachylepis vittata* (Olivier, 1804)	Bridled mabuya	Razmjo et al. (2013)
	*Heremites auratus* Linnaeus, 1758. Reported as *Trachylepis aurata* subsp. *transcaucasica*	Golden grass skink	Razmjo et al. (2013)
	*Trapelus ruderatus* (Olivier, 1804) (*Trapelus lessonae*)	Anderson's agama	Razmjo et al. (2013)
	*Testudo horsfieldii* Gray, 1844	Central Asian tortoise	Javanbakht et al. (2015b)
*Haemaphysalis* sp.	*Trachylepis vittata* (Olivier, 1804)	Bridled mabuya	Razmjo et al. (2013)
	*Heremites auratus* Linnaeus, 1758. Reported as *Trachylepis aurata* subsp. *transcaucasica*	Golden grass skink	Razmjo et al. (2013)
	*Trapelus ruderatus* (Olivier, 1804) (*Trapelus lessonae*)	Anderson's agama	Razmjo et al. (2013)
	*Laudakia nupta* (De Filippi, 1843)	Large-scaled rock agama	Razmjo et al. (2013)
*Rhipicephalus sanguineus* sensu lato Latreille, 1806	*Trachylepis vittata* (Olivier, 1804)	Bridled mabuya	Razmjo et al. (2013)
	*Heremites auratus* Linnaeus, 1758. Reported as *Trachylepis aurata* subsp. *transcaucasica*	Golden grass skink	Razmjo et al. (2013)
	*Trapelus ruderatus* (Olivier, 1804) (*Trapelus lessonae*)	Anderson's agama	Razmjo et al. (2013)
	*Laudakia nupta* (De Filippi, 1843)	Large-scaled rock agama	Razmjo et al. (2013)
*Hyalomma marginatum* Koch, 1844. Reported as *Hyalomma marginatum* subsp. *marginatum*	*Testudo graeca* Linnaeus, 1758	Mediterranean spur-thighed tortoise	Adeli-Sardou et al. (2019)
*Hyalomma rufipes* Koch, 1844. Reported as *Hyalomma marginatum* subsp. *rufipes*	*Testudo graeca* Linnaeus, 1758	Mediterranean spur -thighed tortoise	Adeli-Sardou et al. (2019)
**Mite**			
*Hexidionis agamae* Andre, 1929	*Agama* sp.	Agamas	Vercammen-Grandjean et al. (1970)
*Ophionyssus natricis* (Geravis, 1844)	*Python bivittatus* (Kuhl, 1820)	Burmese python	Amanatfard et al. (2014)
*Geckobiella donnae* Paredes-León, Klompen & Pérez, 2012	*Iguana iguana* (Linnaeus, 1758)	Common green iguana	Sharifzadeh et al. (2016)
*Hirstiella* sp.	*Iguana iguana* (Linnaeus, 1758)	Common green iguana	Tavassoli et al. (2017)
*Schoengastia* sp.	*Lacerta strigata* Eichwald, 1831	Caspian green lizard	(Mariaux et al., 2017)
*Pentidionis agamae* (André, 1929)	*Agama* sp.		Vercammen-Grandjean et al. (1970)
**Miyasis**			
*Calliphora vicina* Robineau-Desvoidy, 1830	*Testudo graeca* Linnaeus, 1758	Mediterranean spur-thighed tortoise	Ahmadiara et al. (2011)
*Musca domestica* Linnaeus, 1758	*Pseudocerastes persicus* (Duméril, Bibron & Duméril, 1854)	Persian horned viper	Dehghani et al. (2012)

∗NS: not stated.

The impact of ticks on reptile health was documented by Abbassian-Lintzen (1960) and Razmjo et al. (2013) who associated the infestation by mites and ticks with anaemia, skin disorders, and transmission of pathogens. Later on, Amanatfard et al. (2014) described a case of dermatitis caused by mite infestation in a reptile owner. More recently, epidemiological studies focused on the distribution of mites in various regions in Iran were done, including correlations of infestation with environmental factors (Orlova et al., 2023; Tavassoli et al., 2017). Myiasis was also reported in snakes (Dehghani et al., 2012) and in *Testudo graeca* (Ahmadiara et al., 2011) in Iran, pointing out the need for early diagnosis and treatment for the protection of reptilian fauna (Table 4).

## 4 Conclusions

This paper offers a comprehensive literature review of parasites in reptiles from Iran, with data collected from 1931 to August 2024. Constant parasite survey in endemic reptiles is important considering several species are of conservation concern. It is worth noting that the exact distribution and host-parasite associations should be known and more studies are needed with a focus on new study areas and different reptile species. Advanced diagnosis techniques are needed for accurate identification as well as for understanding the epidemiology and the potential zoonotic implications. Strategies for reptile health, species conservation, and the *One Health* implications should be developed. Considering the risk of co-endangerment and co-extinction of the parasites and their hosts (Gómez and Nichols, 2013), the conservation of parasites of reptiles in Iran especially those infecting endangered hosts should be thought of as a critical mission.

## CRediT authorship contribution statement

**Alireza Sazmand:** Writing – review & editing, Writing – original draft, Validation, Supervision, Project administration, Methodology, Investigation, Conceptualization. **MohammadParsa Miadfar:** Writing – original draft, Methodology. **Georgiana Deak:** Writing – original draft, Methodology. **Mohammad Babaei:** Writing – original draft, Methodology. **Jairo A. Mendoza-Roldan:** Writing – review & editing. **Domenico Otranto:** Writing – review & editing.

## Declaration of competing interest

The authors declare no conflict of interest.
